# Soybean Whey Bio-Processed Using *Weissella hellenica* D1501 Protects Neuronal PC12 Cells Against Oxidative Damage

**DOI:** 10.3389/fnut.2022.833555

**Published:** 2022-03-08

**Authors:** Liqing Yin, Yongzhu Zhang, Fidelis Azi, Mekonen Tekliye, Jianzhong Zhou, Xiaonan Li, Zhuang Xu, Mingsheng Dong, Xiudong Xia

**Affiliations:** ^1^Institute of Agricultural Product Processing, Jiangsu Academy of Agricultural Sciences, Nanjing, China; ^2^College of Food Science and Technology, Nanjing Agricultural University, Nanjing, China; ^3^Institute of Food Safety and Nutrition, Jiangsu Academy of Agricultural Sciences, Nanjing, China; ^4^Faculty of Chemical and Food Engineering, Bahir Dar Institute of Technology, Bahir Dar University, Bahir Dar, Ethiopia; ^5^School of Food and Biological Engineering, Jiangsu University, Zhenjiang, China

**Keywords:** phenolic profile, antioxidant enzyme, apoptosis rate, nerve cell, beverage

## Abstract

Soybean whey, as a byproduct of soybean industry, has caused considerable concern recently because of its abundant nutrients. To further utilize soybean whey, it was fermented with *Weissella hellenica* D1501, and the neuroprotective potency of this beverage was studied in the present work. The phenolic profile and antioxidant capacity of fermented soybean whey (FSBW) were analyzed. The neuroprotective effects were evaluated based on the hydrogen peroxide-stimulated oxidative damage model in a neural-like cell (PC12). Results demonstrated that soybean whey's phenolic contents and antioxidant activities were markedly improved after fermentation. Glycoside isoflavones were efficiently converted into aglycones by *W. hellenica* D1501. FSBW extract apparently increased cell viability, decreased reactive oxide species levels, and protected antioxidant enzymes in oxidative damage. Furthermore, FSBW effectively reduced apoptosis rate by inhibiting Bax protein and improving Bcl-2 and Bcl-xL proteins. FSBW ameliorated the cell cycle through the decrease of p21 protein and an increase of cyclin A protein. The findings of this study thus suggested that *W. hellenica* D1501-fermented soybean whey could potentially protect nerve cells against oxidative damage.

## Graphical Abstract

Schematic diagram of the potential mechanisms of the neuroprotective effects of FSBW *in vitro*. 
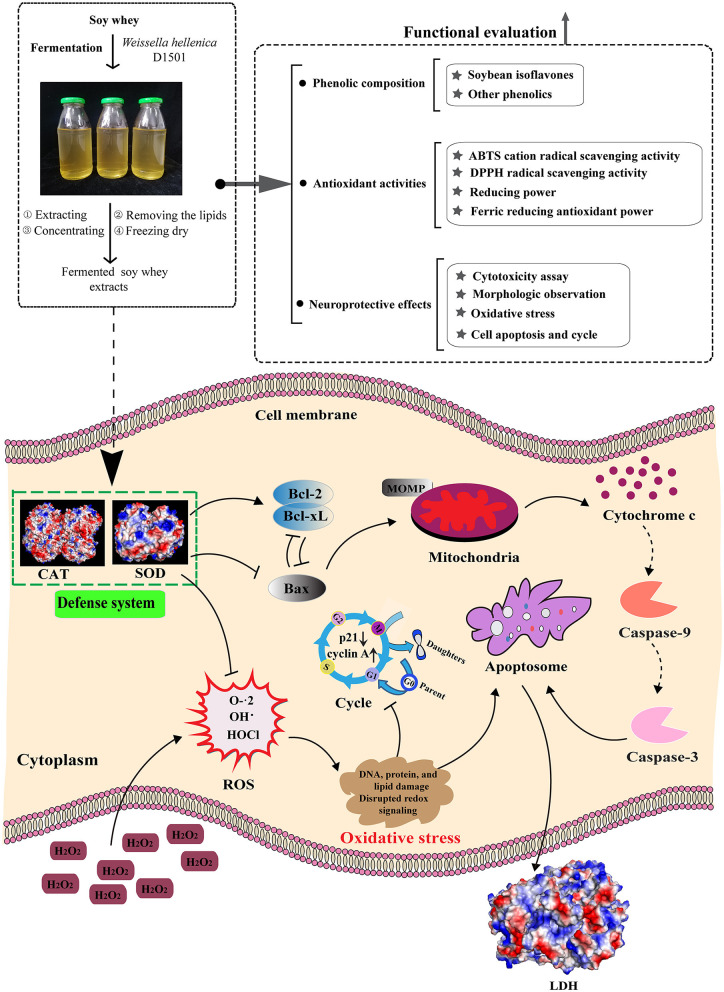

## Introduction

Soybean products are popular worldwide that are linked to various high-quality nutrition and associated health-promoting effects (1, 2). Large quantities of soybean whey are generated during processing soybean-derived food products, and it is generally discarded as waste, resulting in environmental problems (3, 4). Reports have shown that soybean whey is rich in various useful compounds, including phenolics, common isoflavones (glycitin, daidzin, genistin, glycitein, daidzein, and genistein), and oligosaccharides, which benefit human health (5, 6). Instead of treating soybean whey as a waste, it has been utilized as a potential resource in a variety of ways. Soybean whey can be used as a useful substrate for probiotics to produce a beverage with a potential *in vitro* antihypertensive bioactivity (3). Soybean whey bio-processed with *Lactobacillus amylolyticus* can be served as a coagulant for tofu production (6). Soybean whey can also be directly biotransformed into a novel functional alcoholic beverage with high content of free isoflavones after *Saccharomyces cerevisiae*-fermentation (4). Furthermore, isoflavone glycosides (glycitin, daidzin, and genistin) in soybean whey can be efficiently hydrolyzed into its corresponding aglycones (glycitein, daidzein, and genistein) with high bioactivity during fermentation (7–9).

Neurodegenerative diseases have drawn much attention recently due to their damage to the human lifespan (10, 11). The overproduction of reactive oxygen species (ROS) can oxidize biomacromolecules, destroy mitochondrial function, and promote neuronal apoptosis, consequently causing neurodegenerative diseases (12, 13). Plant polyphenols are widely believed to be a potent free radical scavenger to attenuate oxidative stress (14). There is increasing evidence that polyphenolic extracts, especially soybean isoflavones, are effective in the mice model of Alzheimer's and Parkinson's diseases (15, 16). Fermented soybean whey (FSBW) with lactic acid bacteria has been reported to be rich in phenolics, especially aglycone isoflavones (6, 7). Nevertheless, the information involved in the protective effects of lactic acid bacteria-fermented soybean whey on nerve cells and the underlying mechanisms is still unclear.

PC12 cells were derived from rat pheochromocytoma cells and commonly used as an *in vitro* model for studying neuronal apoptosis, oxygen sensor mechanisms, and neuronal differentiation (17). In this study, the neuroprotective effects of soybean whey bio-processed with *W. hellenica* D1501 on preventing hydrogen peroxide (H_2_O_2_)-stimulated oxidative damage were evaluated in PC12 cells. We also identified the phenolic composition and investigated the antioxidant activity of FSBW. The present study aimed to develop FSBW as a potential beverage to protect brain neurons from oxidative damage.

## Materials and Methods

### Bacteria

The strain *W. hellenica* D1501 (Genbank: FJ654461) was first cultured in MRS (pH 6.2) at 37°C for 24 h and then expanded under the same condition for 16 h. The cells were obtained after centrifugation at 5,000 × *g* for 10 min and then resuspended using sterilized physiological saline (0.85% g/v) for soybean whey fermentation.

### Soybean Whey Fermentation and Extraction

Soybean whey was first sterilized under 108°C for 15 min. Then, 1% (v/v) of the sterilized physiological saline containing *W. hellenica* D1501 (OD_600nm_ 1.0) was inoculated into soybean whey. The mixture was cultivated in a constant temperature incubator at 37°C for 24 h to obtain FSBW. After fermentation, FSBW was extracted with 80% (v/v) ethanol in an ultrasonic bath and then centrifuged at 10,000 × *g* for 20 min. Subsequently, the supernatant was filtered using a 0.45 μm syringe filter and treated with hexanes to remove the lipids. The filtrate was freeze-dried at−50°C for further study. The unfermented soybean whey (USBW) was treated under the same conditions except *W. hellenica* D1501 fermentation and served as a control.

### Phenolic Composition and Antioxidant Capacity

Samples were dissolved in 80% (v/v) methanol. A 0.22 μm syringe filter was used to remove solid particles. The phenolic composition was analyzed using high-performance liquid chromatography (HPLC) equipped with the ZORBAX Eclipse Plus C18 reversed-phase analytical column (4.60 × 250 mm, 5 μm, Agilent). 0.4% (v/v) acetic acid in water (buffer A) and acetonitrile (buffer B) were the mobile phases. Detailed elution was conducted as the report of our previous work (18). The total phenolic content (TPC) was measured using Folin-Ciocalteau assay as Xiao et al. (7). The antioxidant capacities, including DPPH radical scavenging activity, ABTS radical cation scavenging activity, reducing power (RP), and ferric reducing antioxidant power (FRAP), were also performed according to Yin et al. (18).

### Influence of USBW and FSBW on Oxidative Damage

#### Cell Culture and Cytotoxicity

PC12 cells were cultivated in DMEM (Gibco, USA) added with streptomycin (100 μg/mL), penicillin (100 U/mL), and fetal bovine serum (10%, Gibco, USA) inactivated by heat. Cells were kept in a 5% CO_2_ incubator at 37°C for at least 2 days before further study. Cell viability was measured according to the method provided by Zhang et al. (19). Briefly, 1.8 × 10^4^ cells were seeded in each well of 96-well plate for 12 h. And then the cells were treated with H_2_O_2_ or the extracts of USBW and FSBW at different concentrations for 4 h. The plate was supplemented with MTT solution and maintained at 37°C for 4 h. Subsequently, the reaction product of MTT was extracted in dimethylsulfoxide (DMSO) (150 μL) after the culture was removed. The optical density was spectrophotometrically read at 490 nm.

#### Influence of USBW and FSBW on H_2_O_2_-Stimulated Loss of Cell Viability

A density of 1.8 × 10^4^ cells/well was seeded in a 96-well plate at 37°C for 12 h and then stimulated with H_2_O_2_ (550 μM) for 4 h and with or without different concentrations of USBW and FSBW extracts. After incubation, cell viability was determined using MTT assay as described above.

#### Influence of USBW and FSBW on LDH Level in Culture Medium

A density of 3.6 × 10^5^ cells/well was seeded in a 6-well plate at 37°C for 12 h. Subsequently, cells were stimulated using H_2_O_2_ (550 μM) and with or without different concentrations of USBW and FSBW extracts for 4 h. According to its instruction, an LDH assay kit (Beyotime, China) was used to determine the LDH levels. Furthermore, the cell morphology of PC12 cells was examined using an inverted microscope (ECLIPSE TE2000-S, Nikon, Japan).

#### Influence of USBW and FSBW on ROS Production

A density of 3.6 × 10^5^ cells/well was seeded in a 6-well plate for 12 h. Subsequently, cells were stimulated using H_2_O_2_ (550 μM) and with or without USBW and FSBW extracts at 3 mg/mL for 4 h. A ROS assay kit (Beyotime, China) was used to measure the ROS level according to its instruction. In brief, the adherent cells were collected using trypsin and then mixed with 10 μM DCFH-DA at 37°C for 20 min. The cells were resuspended in a cell culture medium without serum. The production of ROS was monitored using a flow cytometer (Accuri C6 Plus, BD, USA).

#### Influence of USBW and FSBW on Catalase, Superoxide Dismutase, and Glutathione Peroxidase

After treatment as described above, cells were collected using trypsin and treated with an ultrasonic cell disruption system. After centrifugation at 10,000 × *g* for 20 min, the supernatant obtained was applied to determine antioxidant enzyme activities using catalase (CAT), superoxide dismutase (SOD), and glutathione peroxidase (GSH-Px) kits (Beyotime, China) based on their instructions. The protein content in the lysate was quantified according to the BCA method.

#### Influence of USBW and FSBW on Cell Apoptosis

PC12 cells were treated as above and harvested by trypsin. Cell apoptosis rates were determined based on the instruction of a cell apoptosis kit (Beyotime, China). In brief, cells were washed, resuspended with annexin V-FITC binding buffer, and then incubated with Annexin V-FITC and prodium iodide (1:2, v/v) at room temperature for 20 min. The fluorescence was quantified using a flow cytometer (Accuri C6 Plus, BD, USA).

#### Influence of USBW and FSBW on Cell Cycle

PC12 cells were treated as above and harvested by trypsin. The cell cycle was determined based on the instruction of a cell cycle kit (Beyotime, China). Shortly, PC12 cells were washed and fixed using 70% ethanol (v/v). And then cells were washed again and dyed using a prodium iodide staining reagent. The fluorescence intensity was quantified using a flow cytometer (Accuri C6 Plus, BD, USA).

#### Western Blot Analysis

The western blot analysis was performed following Zhang et al. (19). Shortly, PC12 cells were harvested and lysed using RIPA buffer. The lysate was centrifuged to collect the supernatant. The protein content was quantified with the BCA method. Protein (30 μg/lane) was loaded into 10% or 15% SDS-PAGE gel. After electrophoresis, the protein bands were transferred to a PVDF membrane (Bio-Rad, USA). The membranes were incubated with antibodies against Bax, Bcl-2, Bcl-xL, cyclin A, p21, and β-tubulin proteins. Afterward, the protein signals were enhanced by HRP-conjugated secondary antibody. And then the membrane was treated with an ECL reagent and analyzed by a detection system (ImageQuant LAS4000mini, GE, USA). The densitometric semi-quantifications of the western blot results were determined using ImageJ software.

### Statistical Analysis

All experiments were repeated at least three independent times. Each value was represented as mean ± standard deviation. Data were analyzed by one-way ANOVA (Duncan's *post-hoc* multiple comparisons) in SPSS. The treatment effect was considered significant at *p* < 0.05.

## Results

### Phenolic Compositions of USBW and FSBW

As shown in Table 1, the TPC of soybean whey significantly (*p* < 0.05) increased after *W. hellenica* D1501 fermentation, wherein the TPC of FSBW extract was 18.36% higher than that of USBW. *W. hellenica* D1501 fermentation also showed a significant effect on the phenolic compositions of soybean whey. This study investigated eleven phenolics in soybean whey (Table 1, Supplementary Figure 1). Six soybean isoflavones, including glycitein, genistein, daidzein, glycitin, daidzin, and genistin, were the major phenolic components in soybean whey and accounted for 81.32% of the TPC in USBW and 73.41% of the TPC in FSBW. The glycosidic isoflavones were efficiently transformed into their corresponding aglycones during fermentation. The total glycosidic isoflavone, including glycitin, daidzin, and genistin, was 528.49 mg/100 g extract in USBW, which made up 83.35% of the total soybean isoflavones. The aglycone isoflavone, including glycitein, genistein, and daidzein, was 637.30 mg/100 g extract in FSBW, which made up 94.08% of the total soybean isoflavones. Phenolics like chlorogenic acid, vanillic acid, caffeic acid, ferulic acid, and quercetin were also analyzed by HPLC after fermentation (Table 1). Results showed no effects on chlorogenic acid and vanillic acid, and the others were not detected. Meanwhile, the detected TPC was higher than that obtained from HPLC analysis. This was possibly due to the fact that some phenolic compounds in small concentrations were not detected by HPLC (7).

**Table 1:** Phenolic profile of USBW and FSBW.

**Peak**	**Phenolic compound (mg/100 g)**	**USBW**	**FSBW**
1	Chlorogenic acid	4.69 ± 0.38^Ea^	4.72 ± 0.33^Da^
2	Vanillic acid	16.50 ± 0.96^DEa^	15.26 ± 0.67^CDa^
3	Caffeic acid	N.D.	N.D.
4	Daidzin	318.97 ± 27.75^Aa^	8.62 ± 1.22^Db^
5	Glycitin	65.84 ± 6.54^Ca^	29.03 ± 2.47^Cb^
6	Ferulic acid	N.D.	N.D.
7	Genistin	143.68 ± 9.70^Ba^	2.48 ± 0.12^Db^
8	Daidzein	26.20 ± 2.29^Db^	208.52 ± 24.88^Ba^
9	Glycitein	63.80 ± 7.08^Cb^	236.00 ± 11.50^Aa^
10	Quercetin	N.D.	N.D.
11	Genistein	15.56 ± 1.15^DEb^	192.78 ± 5.90^Ba^
	TPC (mg GAE/100 g)	779.70 ± 31.62^b^	922.86 ± 22.51^a^

*N.D., not detected; TPC, total phenolic content; GAE, gallic acid equivalent. p < 0.05 was considered as the significant level. The different small letters a and b were used to show the significant difference between USBW and FSBW for the same component where “a” indicates a significantly higher yield than “b”. The different capital letters within a column indicate the significant difference and the ordering of results*.

### Antioxidant Properties of USBW and FSBW

**Figure 1:**
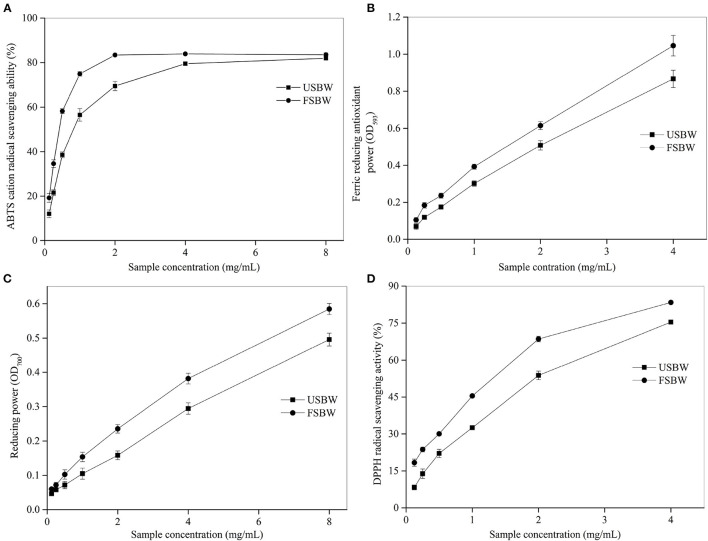
Antioxidant capacity of USBW and FSBW extracts. **(A)** ABTS·^+^ scavenging capacity; **(B)** ferric reducing antioxidant power; **(C)** reducing power; **(D)** DPPH radical scavenging capacity.

The antioxidant activities of foods are up to the composition and concentrations of their antioxidant compounds. Different antioxidants show their radical scavenging activities through different mechanisms (20). Consequently, four assays were chosen to appraise the antioxidant ability of USBW and FSBW in this study. Soybean whey's ABTS·^+^ scavenging ability was significantly enhanced after fermentation (Figure 1A). For instance, when the extract concentration was 0.5 mg/mL, the ABTS·^+^ scavenging ability of FSBW was 58.19%, which was 1.51 times that for USBW. The ABTS·^+^ scavenging ability of USBW and FSBW also exhibited an apparent dose-dependent effect with the increased extract concentration. Furthermore, a similar trend was also observed in FRAP, RP, and DPPH (Figures 1B–D). The half-efficiency concentration (EC_50_) is generally used to express the antioxidant activity of compounds, and its value is inversely related to the antioxidant activity (19). In the present work, EC_50_ values of USBW and FSBW were also analyzed in Supplementary Table 1. The result showed that the EC_50_ values of soybean whey were all significantly decreased after fermentation. The EC_50_ values for ABTS∙^+^ scavenging ability, DPPH scavenging ability, and RP of FSBW, respectively, decreased by 48.91, 40.19, and 20.50% compared with those of USBW.

### Effects of USBW and FSBW on H_2_O_2_-Induced Oxidative Injury

#### Cytotoxicity Evaluation

**Figure 2:**
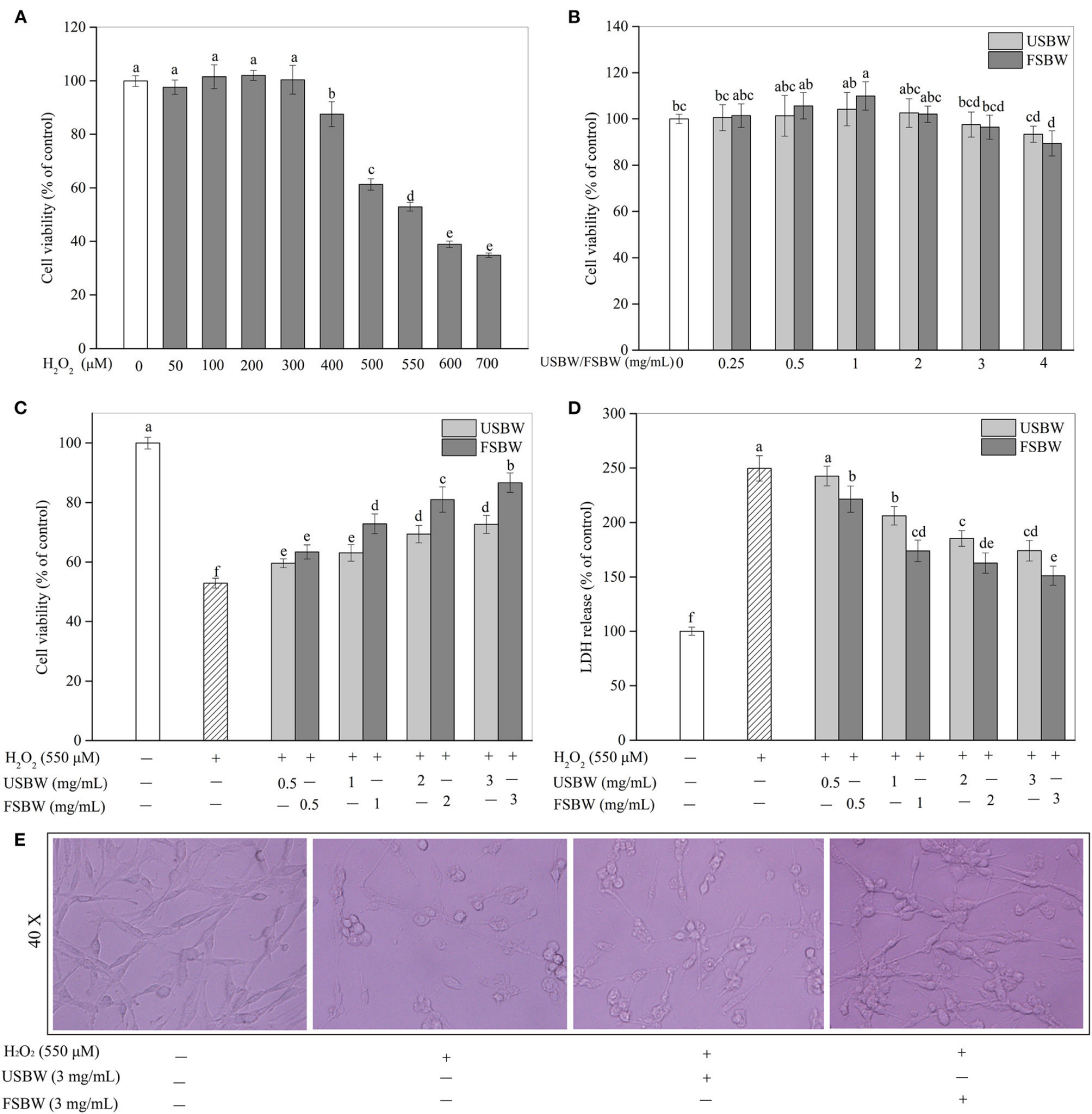
Cytotoxicity and the protection of USBW and FSBW extracts on H_2_O_2_-induced oxidative damage. **(A)** Cytotoxicity of H_2_O_2;_ **(B)** Cytotoxicity of USBW and FSBW extracts; **(C)** Cell viability; **(D)** LDH levels; **(E)** PC12 cell morphology. *p* < 0.05 was considered as the significant level. The small letters were used to indicate the significant difference and the ordering of results.

In this study, PC12 cells were incubated with various concentrations of H_2_O_2_ ranging from 50 to 700 μM for 4 h. As shown in Figure 2A, the H_2_O_2_-induced injury was in a clear dose-dependent manner. The PC12 cell viability held steady when H_2_O_2_ was below 300 μM, and then a rapid decrease was observed (>300 μM). Exposure to 550 μM H_2_O_2_ for 4 h led to a 47.07% decrease in the cell viability of PC12 cells. So 550 μM H_2_O_2_ was chosen to stimulate oxidative injury in the following treatment. The safe dose of the USBW and FSBW extracts was also investigated in this study. The USBW and FSBW extracts below 3 mg/mL were non-cytotoxic to PC12 cells (Figure 2B). However, cell viability exhibited an obvious decrease when PC12 cells were exposed to a higher dose (4 mg/mL) of FSBW extract (Figure 2B). Therefore, the safe dose (<4 mg/mL) of samples for PC12 cells was chosen for further study.

#### Protection of USBW and FSBW on PC12 Cell Viability

Cells were stimulated with 550 μM H_2_O_2_, treated with or without the USBW, and FSBW extracts at nontoxic concentrations (0.5–3 mg/mL) for 4 h. As shown in Figure 2C, cell viability treated with only H_2_O_2_ apparently decreased to 52.93% of the untreated group. However, incubation with soybean whey extracts significantly reduced the cell damage induced by oxidative injury. Additionally, FSBW exhibited higher protection on PC12 cells than USBW. The FSBW extract at 3 mg/mL presented the highest protection on PC12 cells against the H_2_O_2_ damage by increasing the cell viability from 52.93 to 86.66%, which was 1.19 times that of the USBW extract-treated group. Furthermore, USBW and FSBW extracts also significantly reduced the LDH release (Figure 2D) and improved cell morphology (Figure 2E).

#### Attenuation of USBW and FSBW on H_2_O_2_-Induced Oxidative Stress

**Figure 3:**
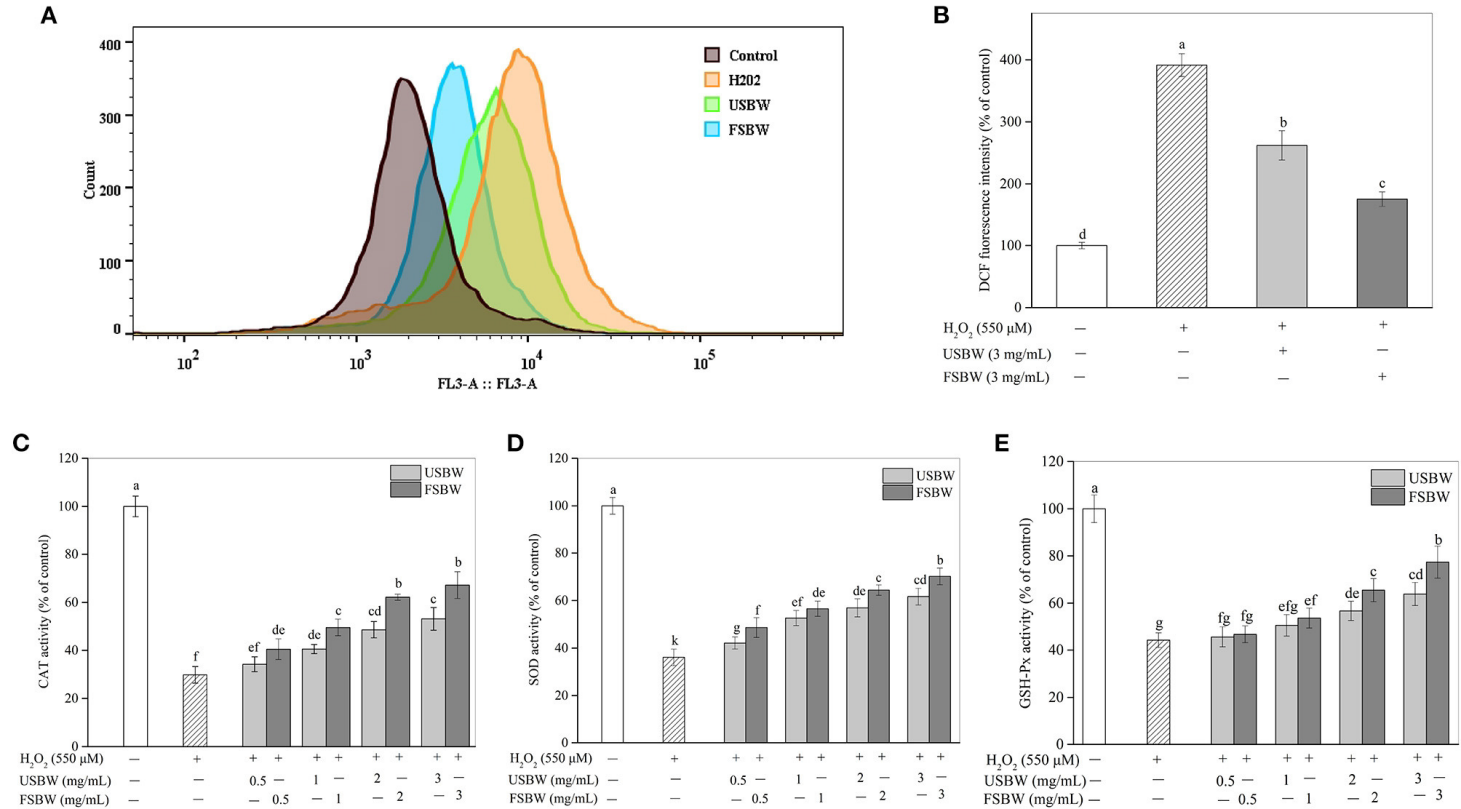
Influence of USBW and FSBW on oxidative stress. **(A)** ROS production; **(B)** Statistical histogram in **(A)**; **(C)** Catalase (CAT) activity; **(D)** Superoxide dismutase (SOD) activity. **(E)** Glutathione peroxidase (GSH-Px) activity. *p* < 0.05 was considered as the significant level. The small letters were used to indicate the significant difference and the ordering of results.

ROS played an essential role in H_2_O_2_-stimulated oxidative damage (21). As shown in Figures 3A,B, H_2_O_2_ treatment resulted in an obvious increase in ROS production in PC12 cells. ROS levels in PC12 cells exposed to only H_2_O_2_ increased by 291.45% of that in the untreated control. However, ROS production was significantly (*p* < 0.05) repressed when PC12 cells were cultured with the USBW and FSBW extracts at 3 mg/mL, decreasing ROS level from 391.45 to 262.10% and 175.40% compared with that of the H_2_O_2_-treated control, respectively. The defense mechanism composed of antioxidant enzymes, such as CAT and SOD, plays a crucial role in preventing oxidative damage caused by ROS (22). As shown in Figures 3C–E, the intracellular CAT, SOD, and GSH-Px activities in PC12 cells stimulated with 550 μM H_2_O_2_ exhibited a significant decrease, only 29.82, 36.10, and 44.27% compared with those in the untreated control, respectively. This effect of H_2_O_2_ on intracellular antioxidant enzyme activities was in agreement with the report of Tang et al. (22). However, treatment with the soybean whey extracts at a range of 0.5 mg/mL-3 mg/mL apparently promoted CAT, SOD, and GSH-Px activities in a dose-dependent way (Figures 3C–E). Furthermore, FSBW showed a more vital ability to induce the production of CAT, SOD, and GSH-Px. For example, the enzyme activities of CAT, SOD, and GSH-Px in the FSBW-treated group at 3 mg/mL were 1.26, 1.14, and 1.21 times those in the USBW-treated group, respectively.

#### Influence of USBW and FSBW on Cell Apoptosis

**Figure 4:**
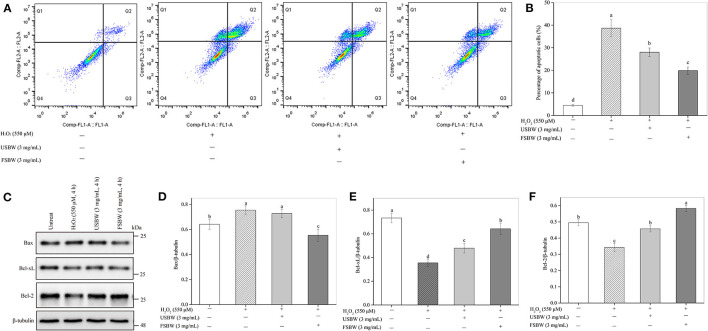
Influence of the USBW and FSBW extracts on H_2_O_2_-induced cell apoptosis. **(A)** Cell apoptosis detected using a flow cytometer; **(B)** Statistical histogram in **(A)**; **(C)** Bcl-xL, Bax, and Bcl-2 levels; **(D–F)** Statistical histogram in **(C)**. *p* < 0.05 was considered as the significant level. The small letters were used to indicate the significant difference and the ordering of results.

As shown in Figure 4, H_2_O_2_ treatment remarkably increased the PC12 cell apoptosis rate. The early and late apoptotic cells, respectively, distributed in Q3 and Q2 regions. The total apoptosis rate of H_2_O_2_-treated cells (38.71%) was 8.66-fold higher than that in the untreated control (4.47%). However, this increase of the PC12 cell apoptosis was significantly attenuated by USBW extract at 3 mg/mL (28.01%), with a 27.64% decrease in the cell apoptosis ratio compared with that in the H_2_O_2_-treated control (Figures 4A,B). FSBW also significantly decreased the cell apoptosis by 48.72% compared with H_2_O_2_-treated control. Furthermore, FSBW showed a stronger ability to decrease PC12 cell apoptosis. The total apoptosis rate in the FSBW-treated group was only 70.87% of that in the USBW-treated control.

The Bcl-2 and Bcl-xL proteins are crucial anti-apoptotic cytokines, and Bax is an apoptosis-promoter closely related to cell death, which usually forms a complex with Bcl-2 (23). To further study the protective mechanisms of USBW and FSBW, Influence on the expression levels of Bcl-2, Bcl-xL, and Bax protein, was investigated. Figures 4C,D showed that H_2_O_2_ treatment significantly (*p* < 0.05) up-regulated the pro-apoptotic protein Bax level. Inversely, the induced decreases by H_2_O_2_ in anti-apoptotic protein levels of Bcl-2 and Bcl-xL were markedly inhibited after USBW or FSBW treatments (Figures 4C,E,F), wherein the Bcl-2 and Bcl-xL protein levels in FSBW-treated cells, respectively, increased by 70.11 and 81.01% (*p* < 0.05). Therefore, FSBW effectively protected PC12 cells against oxidative injury-induced apoptosis by up-regulating the anti-apoptotic protein levels of Bcl-2 and Bcl-xL and down-regulating the pro-apoptotic protein level of Bax.

#### Influence of USBW and FSBW on Cell Cycle

**Figure 5:**
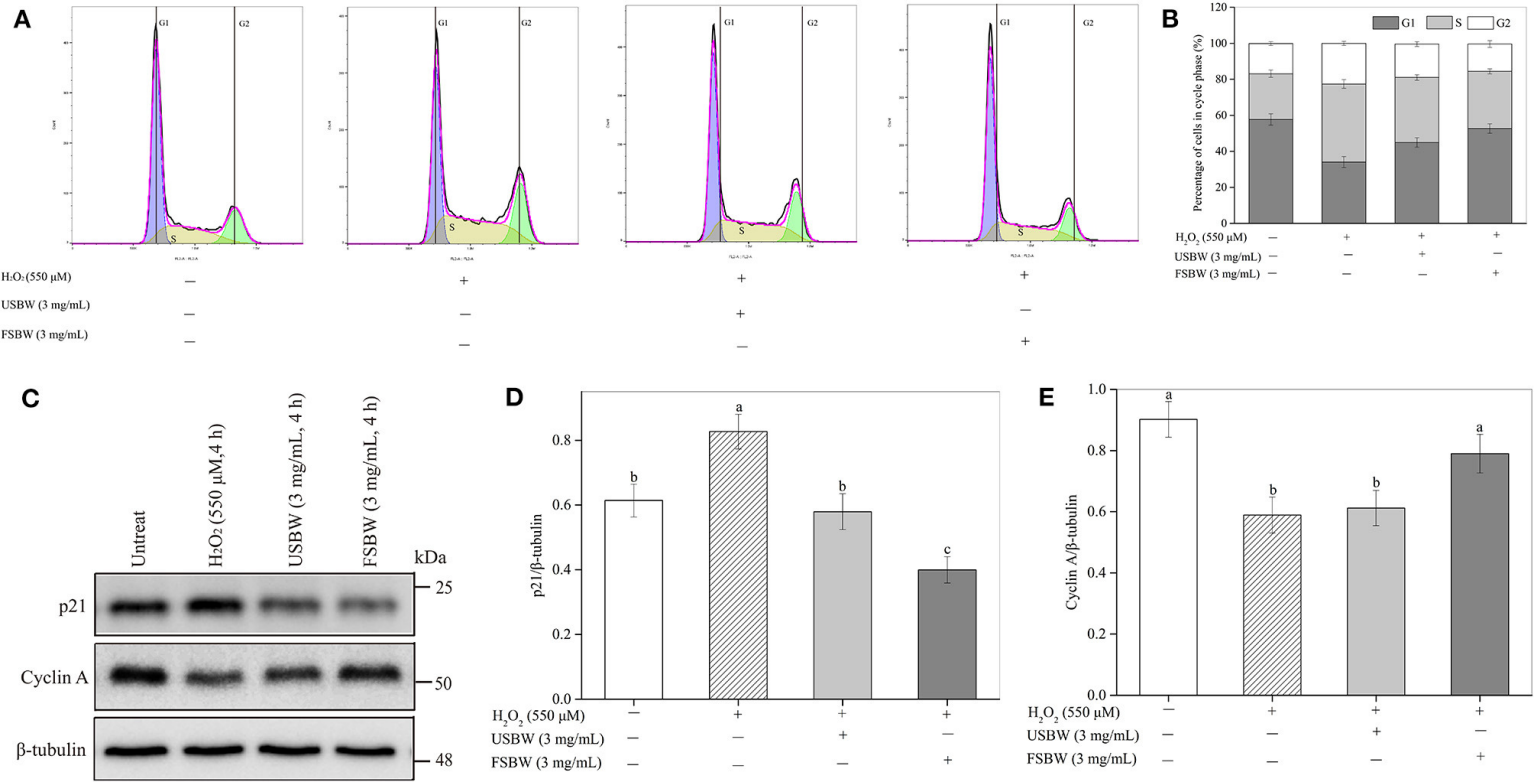
Influence of USBW and FSBW in H_2_O_2_-induced cell cycle arrest. **(A)** Cell cycle detected using a flow cytometer; **(B)** Statistical histogram in **(A)**; **(C)** Cyclin A and p21 levels; **(D,E)** Statistical histogram in **(C)**. *p* < 0.05 was considered as the significant level. The small letters were used to indicate the significant difference and the ordering of results.

As shown in Figures 5A,B, H_2_O_2_ inhibited PC12 cell proliferation by inducing S phase cell cycle arrest. H_2_O_2_ stimulation significantly (*p* < 0.05) increased the cell number at the S phase (43.28%) compared with the untreated control (25.43%), and the G1 phase cell reduced from 57.68% to 34.05%. The cell cycle arrest induced by H_2_O_2_ was inhibited by USBW extract, and this protection was improved after *W. hellenica* D1501 fermentation. For instance, the S phase cell significantly decreased from 43.28 to 36.05% in the USBW-treated group and 31.81% in the FSBW-treated group compared with the H_2_O_2_-treated control, and the G1 phase cell increased from 34.05 to 44.87% and 52.63%, respectively. The important proteins cyclin A and p21 participated in the cell cycle were measured to investigate the cellular mechanisms (Figures 5C–E). The level of cyclin A protein in PC12 cells suffered a significant decrease after H_2_O_2_ treatment, which was reduced by 34.71% compared to control. At the same time, an apparent increase in p21 protein level was also observed in the H_2_O_2_-treated group. The observed S phase cell cycle arrest induced by H_2_O_2_ was significantly attenuated by USBW or FSBW extracts through significant increase of the cyclin A protein level and decrease of the p21 protein level in PC12 cells. FSBW exhibited a more significant inhibitory effect on H_2_O_2_-induced S phase arrest than USBW through improving a higher expression level of cyclin A protein and limiting p21 protein expression.

## Discussion

Soybean foods are a vital source of dietary polyphenols because of their abundant isoflavone content, which mainly exist as glucosides and aglycones (5, 6). It has been reported that isoflavones can ameliorate various diseases, including cardiovascular disease, tumor, and nervous disorder (15, 16, 24). Several reports have described that abundant soybean isoflavones are mostly transferred to soybean whey in the process of soybean foods (6, 7). In this study, the total content of 634.05 mg/100 g extract soybean isoflavones has existed in USBW, which indicated that soybean whey could serve as a good source for isoflavones. However, as a by-product of soybean products in large amounts every year, soybean whey is generally discarded by the food industry, thus resulting in environmental problems (3).

Microbial fermentation has been an efficient strategy to fully utilize soybean whey. Tu et al. (25) showed that soybean whey was a suitable substrate for water kefir to produce a new type of biomedical beverage due to its high phenolic content after fermentation. Chua et al. (4) found that *Saccharomyces cerevisiae*-fermentation can efficiently transform low bioactivity glucoside isoflavone to the corresponding high bioactivity aglycones and enhance the antioxidant capacity of soybean whey, obtaining an alcoholic beverage with fruity and floral notes. In this study, *W. hellenica* D1501 fermentation also significantly affected the phenolic content and composition. TPC in soybean whey significantly (*p* < 0.05) increased after fermentation and most isoflavone glucosides were converted into their corresponding aglycones. Most natural phenolic compounds generally occur as bound forms by forming covalent bonds with sugars and proteins, which are difficult to use for humans (5, 26). The enzymes produced by microorganisms, such as protease, cellulase, and glucosidase, can release bound phenolic acids, which well explains the increased TPC and aglycone isoflavone content of soybean whey after fermentation in this study (18). Plant phenolics exhibit a strong antioxidant ability to scavenge free radicals and form chelate complexes with prooxidant metals (27). Isoflavones in aglycone form exhibit higher antioxidant activity and are absorbed faster by cells than their corresponding glucosides because of their higher lipophilicity and smaller molecular weight (7–9). Therefore, the soybean whey's antioxidant activity, such as the free radical scavenging capacity of ABTS·+ and DPPH and the reduced power on Fe^3+^, significantly (*p* < 0.05) increased after *W. hellenica* D1501 fermentation. Our results demonstrated that *W. hellenica* D1501 fermentation effectively produced a soybean whey beverage with high antioxidant activity.

The overproduction of ROS can oxidize biomacromolecules, break mitochondrial function, and promote neuronal apoptosis, consequently causing neurodegenerative diseases (12). Soybean whey was found to be rich in phenolics, especially isoflavones, which had a strong ability to scavenge free radicals (14). Consequently, the neuroprotective effect of FSBW against oxidative injury in PC12 cells was further studied. The study indicated that H_2_O_2_ caused a strong oxidative injury in PC12 cells by significantly increasing ROS levels. The overproduction of ROS induced by H_2_O_2_ broke down the membrane integrity, caused cell morphological abnormality, damaged antioxidant enzyme, and resulted in cell apoptosis and cell cycle arrest, thereby presenting a significant decrease in PC12 cell viability. Evidence shows that antioxidants can protect cells against various stressors, concluding oxidative stress through their antioxidant action (28). In the present study, we found that soybean whey protected PC12 cells from H_2_O_2_-induced oxidative damage by maintaining cell viability, inhibiting the overproduction of ROS, reducing the loss of antioxidant enzymes, and attenuating cell apoptosis and cell cycle arrest. Furthermore, we found that USBW and FSBW extracts protected PC12 cells from oxidative injury-induced apoptosis through regulation of Bax, Bcl-2 and Bcl-xL proteins. In addition, USBW and FSBW notably reduced the S phase cell cycle by inhibiting the expression of p21 protein and improving the expression of cyclin A protein. Cho et al. (29) demonstrated that the leaf extract with a high phenolic content exhibits a strong antioxidant capacity and can significantly increase PC12 cell viability and membrane integrity, and reduce intracellular oxidative stress in a dose-dependent manner. Im et al. (30) presented that the phenolic compounds in romaine lettuce could help neuronal PC12 cells withstand oxidative stress by maintaining the integrity of cell membrane. Ma et al. (31) indicated that soybean isoflavone genistein could effectively recover PC12 cells' redox balance by decreasing the ROS production induced by Aβ25-35. Liu et al. (32) also found that H_2_O_2_-stimulated damage was effectively attenuated by the flavonoid extract derived from *Rosa laevigata Michx* fruit. Accordingly, the protective effects of soybean whey on PC12 cells were possibly attributed to its abundant phenolic component, especially the isoflavones, which can effectively enhance the ROS scavenging ability of PC12 cells.

The protection of soybean whey on PC12 cells was remarkably promoted by *W. hellenica* D1501 fermentation. This finding might be attributed to the increased phenolics and aglycone isoflavones in soybean whey after fermentation, which exhibited stronger antioxidant activity to scavenge H_2_O_2_-induced ROS, resulting in higher cell viability in the FSBW-treated group than that in the USBW-treated group. This was in agreement with the previous study results that fermented Kudzu root with higher flavonoid content showed stronger bioactivity to protect PC12 cells against H_2_O_2_-induced injury than non-fermented (33). Furthermore, soybean isoflavone glucosides were converted to their corresponding aglycones with high biological activity. The aglycones were more fastly absorbed by the cells culminating in a more effective activation of the antioxidant mechanism to remove ROS in cells (8). Di Cagno et al. (9) also found that fermented soybean milk rich in isoflavone aglycones exhibits higher bioactivity to protect the human intestinal Caco-2 cells against toxin-induced damage. The present study indicated that FSBW could potentially protect nerve cells from oxidative damage, thereby contributing to consumer's health.

In soybean industry, soy whey is a by-product from the preparation of soybean products such as tofu, and soy protein isolate, among others. Large amounts of soy whey are produced every year, and these are generally discarded and considered as waste, which have aggravated burden of the industry on sewage treatment and also a wastage of this resource (34). Based on our findings, these soy whey can be processed into a functional beverage with high antioxidant activity and potential neuroprotective effects by fermentation with *W. hellenica* D1501. Furthermore, this processing method has the advantages of short cycle (not more than 24 h), simple equipment, and low cost, which is very suitable for industrial mass production. Hence, our study provided a good strategy for the full utilization of soybean whey in industry. Of course, the present work was at the initial stage and not ready for trials in humans. Further studies will be performed in a mice model of Alzheimer's disease to explore the potential neuroprotective mechanism of FSBW.

## Conclusions

*W. hellenica* D1501 fermentation was a good strategy to promote the biological activity of soybean whey. The total phenolic content of soybean whey was significantly increased after fermentation, consequently obtaining a higher antioxidant activity. Glycosides soybean isoflavones were efficiently converted to their corresponding aglycones by *W. hellenica* D1501. Soybean whey fermented with *W. hellenica* D1501 could significantly protect PC12 cells from H_2_O_2_-stimulated oxidative injury through enhancing cell viability, inhibiting ROS production, reducing the loss of antioxidant enzyme, attenuating cell cycle arrest, thus repressing cell apoptosis. The present study suggested that soybean whey fermented with *W. hellenica* D1501 could be used as a soybean beverage with potential health-promoting effects.

## Data Availability Statement

The raw data supporting the conclusions of this article will be made available by the authors, without undue reservation.

## Author Contributions

LY and YZ: investigation, methodology, formal analysis, writing-original draft, and visualization. FA and MT: methodology, data curation, and writing-review and editing. JZ, XL, ZX, and MD: resources, validation, and supervision. XX: conceptualization, data curation, funding acquisition, and writing-review and editing. All authors contributed to the article and approved the submitted version.

## Funding

The financial support from Jiangsu Agriculture Science and Technology Innovation Fund (Grant No. CX(21)2003), Yangzhou Science and Technology Plan (Grant No. YZ2020044), and National Natural Science Foundation of China (Grant No. 31501460) are gratefully acknowledged.

## Conflict of Interest

The authors declare that the research was conducted in the absence of any commercial or financial relationships that could be construed as a potential conflict of interest.

## Publisher's Note

All claims expressed in this article are solely those of the authors and do not necessarily represent those of their affiliated organizations, or those of the publisher, the editors and the reviewers. Any product that may be evaluated in this article, or claim that may be made by its manufacturer, is not guaranteed or endorsed by the publisher.

## Abbreviations

USBW: Unfermented Soybean Whey
FSBW: Fermented Soybean Whey
HPLC: High-Performance Liquid Chromatography
ABTS, 2: 2-azinobis(3-ethylbenzothiazoline-6-sulfonic acid) diammonium salt
DPPH, 2: 2-diphenyl-1-picrylhydrazyl
RP: Reducing Powder
FRAP: Ferric Reducing Antioxidant Power
TPC: Total Phenolic Content
H_2_O_2_: hydrogen peroxide
LDH: Lactic Dehydrogenase
MTT, 3-(4, 5-dimethylthiazol-2-yl)-2: 5-diphenyl tetrazolium bromide
CAT: catalase
SOD: Superoxide Dismutase
ROS: Reactive Oxygen Species
DMEM: Dulbecco's modified Eagle's Medium
GAE: Gallic Acid Equivalent
GSH-Px: glutathione peroxidase.
