# Supplementary material for: Soybean Whey Bio-Processed Using Weissella hellenica D1501 Protects Neuronal PC12 Cells Against Oxidative Damage
Source: Front Nutr. 2022 Mar 8;9:833555. doi: 10.3389/fnut.2022.833555 (PMC8957961; doi:10.3389/fnut.2022.833555)
Supplement: Supplementary file 1 [file Data_Sheet_1.docx]

**Figure legends**

**Supplementary Figure 1** HPLC chromatograms of phenolics in soy whey after different treatment. **(A)** a mixture of 11 standards; **(B)** the phenolics in USBW; **(C)** the phenolics in FSBW. 1, chlorogenic acid; 2, vanillic acid; 3, caffeic acid; 4, daidzin; 5, glycitin; 6, ferulic acid; 7, genistin; 8, daidzein; 9, glycitein; 10, quercetin; 11, genistein. USBW, unfermented soy whey. FSBW, fermented soy whey.

**
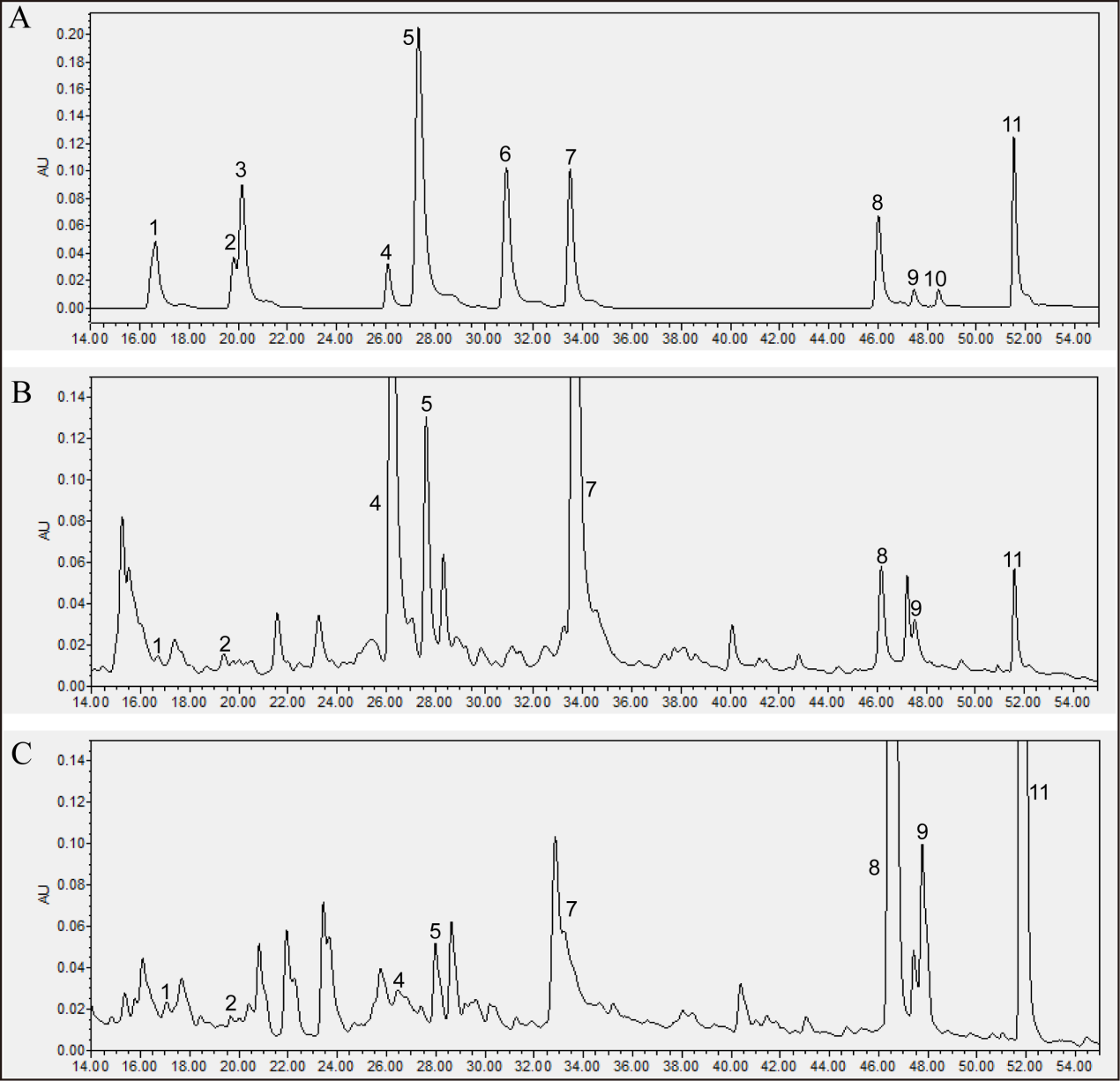
**

**Supplementary Figure 1**

**Supplementary Table 1** Half-efficiency concentration (EC_50_, mg extract/mL) of USW and FSW extracts.

| Samples | ABTS^+^ scavenging | Reducing powder | DPPH scavenging capacity |
| --- | --- | --- | --- |
|  |  |  |  |
| USBW | 0.92±0.73^a^ | 8.05±0.28^a^ | 2.09±0.13^a^ |
| FSBW | 0.47±0.22^b^ | 6.40±0.56^b^ | 1.25±0.09^b^ |

^*^ EC_50_ was the effective concentration of the extracts that removed 50% initial DPPH radical, 50% initial ABTS·^+^ and the absorbance 0.5 for reducing power, respectively. EC_50_ value was obtained by interpolation or extrapolation from linear regression analysis of the data obtained with dose-response effect (Xiao et al., 2015). Means with different small letters within a column were significantly different (*p* < 0.05). Values were presented as mean ± SD (n = 3).
